# Temporal Trends and Outcome of Patients with Acute Coronary Syndrome and Prior Myocardial Infarction

**DOI:** 10.3390/jcm10235580

**Published:** 2021-11-27

**Authors:** Katia Orvin, Alon Shechter, Doron Zahger, Vitaly Shklovski, Tal Ovdat, Roy Beigel, Ran Kornowski, Alon Eisen

**Affiliations:** 1 Rabin Medical Center, Cardiology Department, The Faculty of Medicine, Tel-Aviv University, Tel Aviv 69978, Israel; alonshechter@gmail.com (A.S.); ran.kornowski@gmail.com (R.K.); alon201273@gmail.com (A.E.); 2 Soroka University Medical Center, Cardiology Department, Faculty of Health Sciences, Ben Gurion University of the Negev, Beer Sheva 84105, Israel; dzahger@bgu.ac.il; 3 Barzilai University Medical Center, Cardiology Department, Ben-Gurion University, Ashkelon 78281, Israel; vitalis@barzi.health.gov.il; 4 Sheba Medical Center, Department of Cardiology, The Faculty of Medicine, Tel-Aviv University, Tel Aviv 69978, Israel; Tal.Cohen@sheba.health.gov.il (T.O.); Roy.Beigel@sheba.health.gov.il (R.B.)

**Keywords:** temporal trends, acute coronary syndrome, recurrent cardiovascular events, ACSIS

## Abstract

Patients who have previously had a myocardial infarction (MI) are considered a high-risk group with increased risk for cardiovascular (CV) events. During the last decade, the outcome of acute coronary syndrome (ACS) patients has improved due to advances in medical therapy and interventional techniques. We aimed to examine temporal trends and outcomes of patients with prior MI admitted due to ACS from the Acute Coronary Syndrome Israeli Survey (ACSIS). Included were 16,934 ACS patients, of whom 31.4% had prior MI. For temporal trend analysis, the cohort was divided into an early period (2000–2008) and late period (2010–2018). For patients with prior MI, patients in the late period had a higher rate of CV risk factors and were treated more frequently with revascularization and guidelines-directed medical therapy. Recurrent MI (6.7% vs. 12%, *p* < 0.001), MACE (10.6% vs. 21%, *p* < 0.001) and 1-year mortality (10.7% vs. 14.6%, *p* < 0.001) were significantly lower in the late period. However, the mortality rate for patients with prior MI remained higher compared with patients without prior MI (10.7% vs. 6.8% *p* < 0.001) with an overall higher mortality rate in the STEMI group. Thus, despite significant improvement in outcome measures in the contemporary era, ACS patients with prior MI are still at increased risk for recurrent ischemic CV events and mortality.

## 1. Introduction

Patients who have previously had a myocardial infarction (MI) are at increased short and long-term risk for cardiovascular (CV) events, including recurrent MI and CV mortality [1,2,3,4,5,6,7]. About 20% of patients with prior MI experience subsequent ischaemic events beyond the first year following the initial cardiac event [1]. Secondary prevention programs, intensified follow up, long term medical treatment, and cardiac rehabilitation programs are mandatory to reduce the risk of recurrent CV events.

During the last decade, there has been a rapid and significant improvement in the prognosis of patients with ACS due to advancements in drug therapy and interventional techniques [8,9,10]. However, it is not well established whether similar trends exist in ACS patients who had a prior MI, a particularly high-risk group.

Therefore, we aimed to examine temporal trends in the prevalence, characteristics, treatment strategies, and clinical outcomes of patients with prior MI who were admitted to Israeli medical centres due to ACS.

## 2. Material and Methods

The present study is a retrospective cohort data analysis of patients from the Acute Coronary Syndrome Israeli Survey (ACSIS) carried out between the years 2000–2018. Details of the national registry have been previously reported [11]. In brief, ACSIS is a biennial prospective national registry of all patients with ACS hospitalized in 25 coronary care units and cardiology departments in all general hospitals in Israel over a 2-month period (March to April). Clinical, historical, and demographic data were recorded on prespecified forms for all admitted patients diagnosed with ACS. Admission and discharge diagnoses were recorded by the attending physicians based on electrocardiographic, clinical, and biochemical criteria. Patient management was at the discretion of the attending physicians according to clinical judgement and based on contemporary guidelines. All patients provided informed consent and each institution received the approval of its institutional review board. Patients and the public were not involved in any way in the study.

All patients enrolled in the ACSIS registry between 2000 and 2018 were included in the present study. In order to investigate temporal trends, the study population was divided into an early period (2000–2008) and a late period (2010–2018).

Clinical outcomes included 30-day recurrent MI, 30-day major adverse cardiac events (MACE), which was a composite of death, ACS, stroke, unstable angina, stent thrombosis, and urgent revascularization. In addition, 30-day and 1-year mortality were recorded. Data for 30-day MACE were ascertained and adjudicated by hospital chart review, telephone contact, and clinical follow-up data. Mortality data at 30 days were determined for all patients from hospital charts and by matching identification numbers of patients with the Israeli National Population Register. One-year mortality data were ascertained through the Israeli National Population Registry.

### Statistical Analysis

Patients’ characteristics are presented as *n* (%) for categorical variables, and as mean (standard deviation) for continuous variables. A chi-square test was used for comparison of categorical variables, and t-test for continuous variables. Survival curves are presented, and the Kaplan–Meier log rank test was used to test the impact of the variable of interest on survival.

To reduce confounding between the early and late period, a propensity score matching was performed. The propensity score evaluates the chance for being in the late period, given the following variables: age, sex, dyslipidaemia, hypertension, diabetes mellitus, chronic kidney disease, history of congestive heart failure, prior peripheral vascular disease, prior coronary artery bypass (CABG) surgery, prior percutaneous coronary intervention (PCI), active smoking, and family history of coronary heart disease. Missing values in these variables were <7%, and were imputed with “NO” or mean as appropriate. A 1:1 matching was conducted among MI patients (area under the curve (AUC) of the model was 0.71) and among non-MI patients (AUC was 0.61), with a calliper of 0.05. All tests were conducted at a two-sided overall 5% significance level (≤0.05).

## 3. Results

### 3.1. Prior MI vs. No Prior MI

Between 2000–2018, 16,934 patients were enrolled in the ACSIS registry, of whom 5317 (31.4%) had a prior MI. Patients with prior MI were older, had more frequent CV risk factors and CV morbidities such as peripheral vascular disease (14% vs. 5.7%, *p* < 0.001) and cerebrovascular disease (11.9% vs. 6.5% *p* < 0.001) but were less likely to be active smokers (Supplementary Material Table S1). Furthermore, before the index admission, patients with prior MI were more frequently receiving guidelines-directed medical therapy including aspirin (80.6% vs. 32.7%, *p* < 0.001), ACE-inhibitors/ARB’S (65% vs. 33.5% *p* < 0.001), beta blockers (55.3% vs. 20.7%, *p* < 0.001), and statins (61.9% vs. 28.6%, *p* < 0.001) (Supplementary Materials Table S1). Patients with prior MI presented more commonly with non-ST elevation ACS (NSTE-ACS) rather than ST elevation MI (STEMI) (Supplementary Material Table S1), and acute heart failure symptoms (Killip III and IV) and suffered more frequently from in-hospital MI-related complications. However, patients with prior MI less frequently underwent coronary revascularisation procedures (58.2% vs. 69.1% respectively, *p* < 0.001). Although patients with prior MI were more likely to be discharged with guidelines-directed medical therapy, they were referred less frequently in cardiac rehabilitation programs (44% vs. 52.2% *p* < 0.001). Regarding outcome, patients with prior MI suffered more frequently from 30-day re-MI/angina (8.9% vs. 6.4%, *p* < 0.001), 30-d MACE (16.2% vs. 13.2%, *p* < 0.001), and 30 day and 1-year mortality (5.6% vs. 4.5%, *p* = 0.003 and 12.8% vs. 8.2%, *p* < 0.001, respectively) (Supplementary Material Table S1).

### 3.2. Early vs. Late Period in Patients with Prior MI

**Figure 1:**
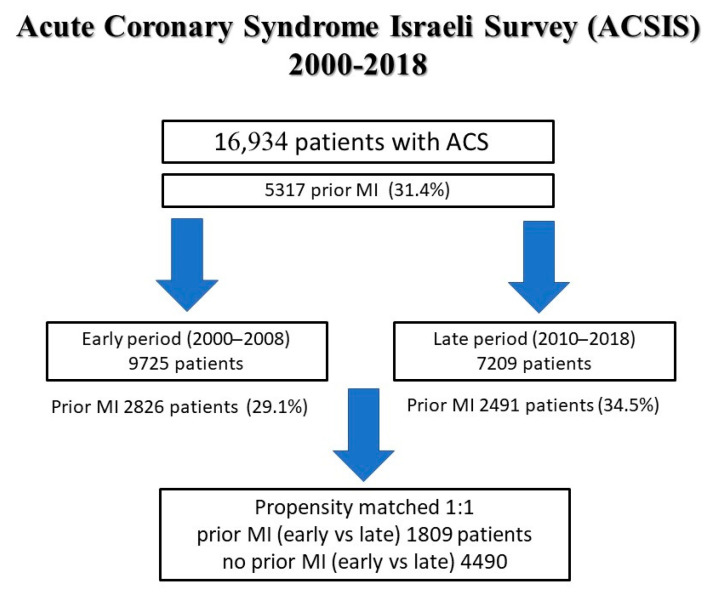
Study flow chart.

Temporal trends were analysed and compared between the early period (2000–2008) and the late period (2010–2018) (Figure 1). Patients with prior MI in the late period had a higher risk profile and were treated more frequently with guidelines-directed medical therapy post MI (Table 1). Moreover, they had undergone more previous coronary revascularization procedures compared with their counterparts in the earlier period (83.2% vs. 57.9%, *p* < 0.001) (Table 1). In addition, in the late period, patients with prior MI presented more frequently with NSTE-ACS (71.5% vs. 65.2%, *p* < 0.01) and less with acute heart failure symptoms (Killip III and IV) (Table 2). In-hospital complications were significantly less frequent in the late period (Table 2). Patients with prior MI in the late period were more frequently discharged with guidelines-directed medical therapy including P_2_Y_12_ inhibitors (88.4% vs. 56.5%, *p* < 0.001), ACE-inhibitors/ARB’S (76% vs. 38.1% *p* < 0.001), beta blockers (85.2% vs. 79.8%, *p* < 0.001), and statins (96.4% vs. 78.5%, *p* < 0.001) (Table 2). Referral to cardiac rehabilitation programs significantly improved in the late period (49.6% vs. 33.2% *p* < 0.001) (Table 2).

### 3.3. Clinical Outcomes

**Figure 2:**
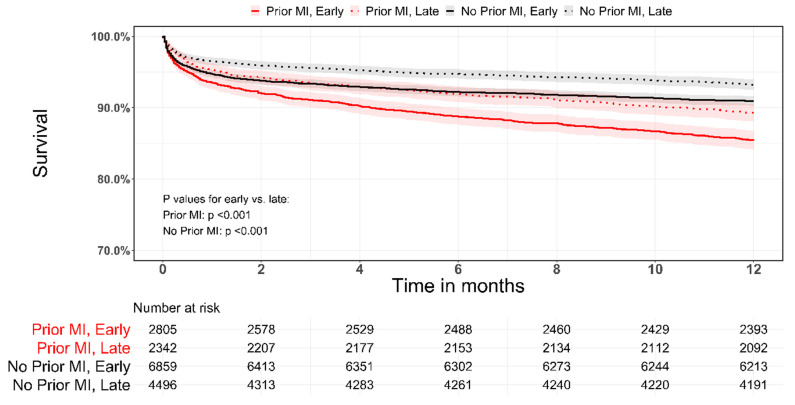
Kaplan–Meier survival curves stratified by prior MI and by time periods (early/late). Log rank with pairwise comparisons with Holm’s adjustment to *p*-value.

Recurrent-MI, 30-day MACE, 30-day and 1-year mortality were significantly lower in the late period for patients with or without prior MI (Table 3, Figure 2). However, patients with prior MI remained with a higher 1-year mortality rate compared with patients without prior MI (10.7% vs. 6.8% *p* < 0.001).

A propensity matched analysis between the early and late periods for patients with prior MI and no prior MI was performed. The resultant matched sample consisted of 1809 matched pairs for prior MI and 4490 pairs for no prior MI. Variables used in the model and baseline characteristics of the matched groups are presented in Supplementary Materials Table S2 and Table 4. In the matched sample of patients with prior MI, the 30-day rates of re-MI/angina and MACE were significantly reduced in the late period (11.2% vs. 6.4%, *p* < 0.001 and 19.7% vs. 11.3%, *p* < 0.001, respectively) (Table 4). However, there was no difference in 30-day and 1-year mortality (Table 4). In contrast, on propensity matching in patients without prior MI, re-MI, 30-day MACE, and 1-year mortality decreased over time.

### 3.4. STEMI and NSTE-ACS

**Figure 3:**
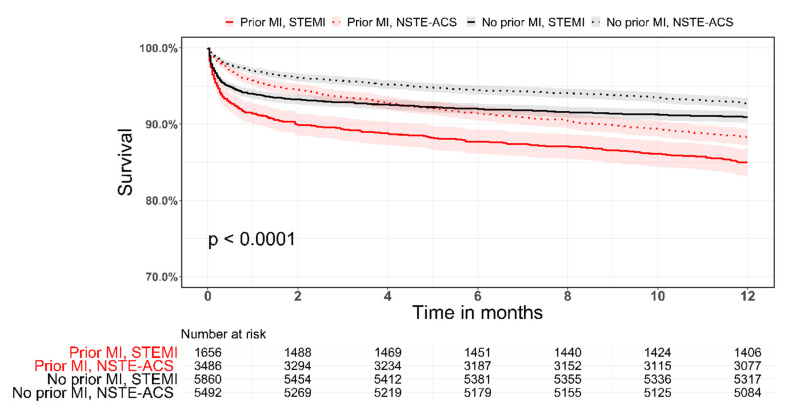
Kaplan–Meier survival curves stratified by prior MI status and by type of index ACS (STEMI/NSTE-ACS).

In patients with prior MI, those who presented with NSTE-ACS had more CV risk factors and comorbidities compared with STEMI patients (Supplementary Materials Table S3). These patients were treated more frequently with guidelines-directed medical therapy (Supplementary Materials Table S3). Both 30-day and 1-year mortality were highest among patients who presented with prior MI and STEMI (Supplementary Materials Table S3 and Figure 3).

Temporal trends in CV risk factors, comorbidities, hospital course, and in-hospital management were similar to the entire cohort (Tables S4 and S5). Recurrent MI and MACE, as well as 1-year mortality were significantly reduced for both STEMI and NSTE-ACS patients in the late period (Table 5) but with an overall higher mortality rate in the STEMI group.

## 4. Discussion

The current study investigated temporal trends among patients with prior MI from a large multicentre ACS registry and found several important findings as follows: (1)~1/3 of patients with ACS had a history of prior MI, most of them presented with NSTEMI-ACS, (2) this high-risk group had higher rates of CV events and mortality up to one year of follow up, (3) despite improved ACS management over time with more invasive coronary revascularization procedures, guidelines-directed medical treatment, and rehabilitation programs, patients with prior MI still had worse CV outcomes, and (4) after a propensity matching statistical adjustment, mortality rates of patients with prior MI remained unchanged over time, and finally, the worst outcome was observed in those prior MI patients presenting with STEMI.

Patients who have had a prior MI are at increased risk for recurrent CV events [1,2,3,4,5,6,7]. In recent years, significant efforts have been invested to improve treatment modalities and secondary prevention measures in order to reduce recurrent ischemic risk. Extensive research has also been conducted in this high-risk population concerning potent antiplatelet therapy, which demonstrated a greater ischemic risk reduction with more “aggressive” treatment [2,3,4,5]. In this study of ACS patients, the use of guidelines-directed medical therapy during hospitalization and at discharge was significantly improved over time for patients with and without prior MI. Furthermore, coronary revascularization was performed more frequently during the index event. These significant changes over time reflect the evolution of ACS guidelines and practice in accordance with accumulated data. Accordingly, we observed a significant reduction in recurrent ischemic events (recurrent MI and 30-day MACE) and mortality in the late period. Nevertheless, despite overall reduction in MACE over time, patients with prior MI had a numerically higher event rate than patients without prior MI. The same trend was observed for both NSTE-ACS and STEMI patients. Prior studies assumed that insufficient secondary prevention at admission combined with suboptimal in-hospital treatment may contribute to the dismal outcome of high-risk patients with prior MI. However, our study shows that despite improved adherence to guidelines recommendations, this high-risk group still sustained more frequently recurrent ischemic events and mortality.

Following propensity matching between time periods in patients with prior MI, recurrent MI and 30-day MACE decreased, but short- and long-term mortality remained unchanged. However, in patients without prior MI, all outcomes have improved during the time period. This observation highlights a residual mortality risk for patients with prior MI who are admitted with an ACS. This might be explained by the matched cohort that accounted for variables such as dyslipidaemia (statin use) and prior PCI, which might have an impact on mortality, particularly in patients with prior MI.

Although more likely to receive guidelines-directed medical therapy for secondary prevention, prior MI patients in the late period were still undertreated. Only 80% of such patients were treated with statins, 67.7% with beta blockers, and 79.2% with aspirin. Similarly, Shen L et al. [7] demonstrated suboptimal treatment with secondary prevention medications for patients with prior MI (both NSTEMI and STEMI) in the chronic setting. Our study, which represents a more contemporary cohort, still shows a residual gap in secondary prevention for this high-risk population. Since we demonstrated an improvement in in-hospital care for patients with prior MI in the late period, one can assume that adherence to treatment and post discharge care are the main reasons for the remaining gap, however, this should be further investigated in dedicated studies.

Patients with prior MI who presented with STEMI had the highest short and long-term mortality rate. This supports prior studies which showed that prior MI was an independent predictor of in-hospital mortality even after adjustment for heart failure [7]. These patients deserve more attention and should receive close observation during the post discharge period. Measures should be enhanced to integrate this high-risk group into intensive follow up and rehabilitation programs.

Our study has several limitations. First, this is a retrospective study with inherent disadvantages of an observational study. However, this a multicentre registry that represents all-comers of ACS patients to all hospitals who treat MI in Israel and data were prospectively collected. Second, the time of prior MI was unknown. The time period of recurrent MI could be very broad, from months to years with subsequent different implications on outcome and on adherence to medications [12,13]. In addition, data concerning left ventricular function at follow up, cause of mortality, and actual participation in rehabilitation programs, were unavailable.

## 5. Conclusions

Despite significant improvement in secondary prevention and in hospital care in the contemporary era, patients with prior MI who present with an ACS are at increased risk for recurrent ischemic events and mortality. While 30-day MACE has decreased over time among these patients, increased mortality has remained unchanged.

## Figures and Tables

**Table 1:** Baseline characteristics of patients with and without prior MI, compared between time periods (early 2000–2008 vs. late 2010–2018).

	Prior MI			No Prior MI		
	Early	Late	*p* Value	Early	Late	*p* Value
	*n* = 2826	*n* = 2491		*n* = 6899	*n* = 4718	
Age (years)	66.7 ± 12.6	66.6 ± 12.3	0.829	62.6 ± 13.2	62.7 ± 12.9	0.518
Gender (male)	2244 (79.4)	2072 (83.2)	0.001	5178 (75.1)	3579 (75.9)	0.334
Dyslipidemia	2050 (72.5)	2208 (88.6)	<0.001	3649 (53.0)	3089 (65.7)	<0.001
Hypertension	1852 (65.5)	2025 (81.3)	<0.001	3467 (50.3)	2718 (57.8)	<0.001
Active smoker	805 (28.5)	896 (36.0)	<0.001	2654 (38.5)	1959 (41.5)	0.002
Diabetes mellitus	1186 (42.0)	1279 (51.3)	<0.001	2043 (29.6)	1598 (33.9)	<0.001
Prior CABG	697 (24.7)	554 (22.2)	0.041	300 (4.3)	110 (2.3)	<0.001
Prior PCI	1637 (57.9)	2072 (83.2)	<0.001	691 (10.0)	378 (8.0)	<0.001
Chronic kidney disase	519 (18.4)	464 (18.6)	0.944	476 (6.9)	390 (8.3)	0.007
PVD	432 (15.3)	312 (12.5)	0.004	449 (6.5)	212 (4.5)	<0.001
Stroke/TIA	320 (11.4)	307 (12.3)	0.332	450 (6.5)	304 (6.4)	0.876
History of heart failure	564 (20)	457 (18.3)	0.146	204 (3.0)	143 (3.0)	0.861
Baseline medications						
Aspirin	1880 (82.1)	1873 (79.2)	0.013	1811 (32.3)	1468 (33.2)	0.339
P_2_Y_12_ inhibitors	356 (15.6)	629 (27.8)	<0.001	146 (2.6)	229 (5.3)	<0.001
ACE-I/ARB	630 (57.2)	1468 (69.2)	<0.001	739 (27.4)	1539 (37.6)	<0.001
Beta blockers	1413 (62.0)	1526 (67.7)	<0.001	1361 (24.2)	1038 (24.8)	0.699
Statins	1467 (64.4)	1822 (81.9)	<0.001	1590 (28.3)	1731 (45.3)	<0.001

Values are presented as number (%) or mean ± standard deviation. ACE-I—angiotensin-converting enzyme inhibitor; ARB—angiotensin receptor blocker; CABG—coronary artery bypass graft; PCI—percutaneous coronary; PVD—peripheral vascular disease; and TIA—transient ischemic attack.

**Table 2:** Clinical presentation and management of patients with and without prior MI compared between time periods (early 2000–2008 vs. late 2010–2018).

	Prior MI			No Prior MI		
	Early	Late	*p* Value	Early	Late	*p* Value
	*n* = 2826	*n* = 2491		*n* = 6899	*n* = 4718	
ST elevation at presentation	983 (34.8)	711 (28.5)	<0.001	3743 (54.3)	2250 (47.7)	<0.001
Admission Killip III/IV	306 (10.8)	154 (6.6)	<0.001	425 (6.2)	202 (4.5)	<0.001
Revascularization therapy						
Primary PCI	563 (19.9)	386 (21.4)	0.048	2426 (35.2)	1382 (38.1)	<0.001
Any PCI	918 (58.9)	1545 (62.0)	0.053	2809 (73.1)	3460 (73.3)	0.836
CABG	111 (7.0)	89 (3.6)	<0.001	256 (6.8)	264 (5.6)	0.021
Number of diseased vessels						
1	245 (18.7)	497 (24.2)	<0.001	1277 (36.4)	1591 (37.8)	0.031
2	456 (34.9)	635 (30.9)		1152 (32.8)	1275 (30.3)	
3	573 (43.8)	847 (41.2)		908 (25.9)	1091 (25.9)	
In-hospital complications						
Pulmonary edema (Killip-3)	324 (11.5)	150 (6.0)	<0.001	501 (7.3)	135 (2.9)	<0.001
Cardiogenic shock (Killip-4)	136 (4.8)	67 (2.7)	<0.001	233 (3.4)	138 (2.9)	0.189
Re-MI	66 (2.4)	25 (1.0)	<0.001	101 (1.5)	31 (0.7)	<0.001
Stent thrombosis	19 (1.6)	18 (0.7)	0.017	18 (0.7)	24 (0.5)	0.433
Free wall rupture	6 (0.2)	1 (0.0)	0.176	46 (0.7)	6 (0.1)	<0.001
MR moderate—severe	84 (3.0)	39 (1.6)	0.001	137 (2.0)	65 (1.4)	0.016
Sustained VT (>125 bpm)	58 (2.1)	32 (1.3)	0.037	105 (1.7)	53 (1.1)	0.081
Primary VF	55 (2.0)	25 (1.0)	0.006	156 (2.3)	76 (1.6)	0.016
Acute renal failure	251 (8.9)	155 (6.2)	<0.001	393 (5.7)	214 (4.5)	0.006
Treatment at discharge						
Aspirin	2540 (92.4)	2305 (95.2)	<0.001	6318 (93.7)	4441 (96.3)	<0.001
P_2_Y_12_ inhibitors	1526 (56.5)	2133 (88.4)	<0.001	4191 (62.7)	4098 (89.1)	<0.001
Statins	2144 (78.5)	2315 (96.4)	<0.001	5160 (76.8)	4361 (95.7)	<0.001
ACE/ARBs	1077 (38.1)	1894 (76.0)	<0.001	2436 (35.3)	3490 (74.0)	<0.001
Beta blockers	2183 (79.8)	1967 (85.2)	<0.001	5246 (77.9)	3509 (80.1)	0.007
Referral to cardiac rehabilitation	350 (33.2)	1007 (49.6)	<0.001	1047 (42.2)	2269 (58.7)	<0.001

Values are presented as number (%) or mean ± standard deviation. ACE-I—angiotensin-converting enzyme inhibitor; ARB—angiotensin receptor blocker; CABG—coronary artery bypass graft; PCI—percutaneous coronary; MI—myocardial infarction; MR—mitral regurgitation; VF—ventricular fibrillation; and VT—ventricular tachycardia.

**Table 3:** Clinical outcomes of patients with and without prior MI compared between time periods (early 2000–2008 vs. late 2010–2018).

	Prior MI			No Prior MI		
	Early	Late	*p* Value	Early	Late	*p* Value
	*n* = 1809	*n* = 1809		*n* = 4490	*n* = 4490	
Re MI/Angina	96 (11.2)	83 (6.4)	<0.001	151 (8.6)	128 (4.2)	<0.001
MACE * (30 days)	356 (19.7)	203 (11.3)	<0.001	696 (15.5)	396 (8.9)	<0.001
30-day mortality	89 (4.9)	91 (5.1)	0.860	228 (5.1)	146 (3.3)	<0.001
1-year mortality	218 (12.1)	188 (10.9)	0.272	396 (8.9)	288 (6.7)	<0.001

* A composite of death, ACS, stroke, unstable angina, stent thrombosis, and urgent revascularization. MACE—major adverse cardiac events; MI—myocardial infarction.

**Table 4:** Clinical outcomes of patients with and without prior MI between time periods (early 2000–2008 vs. late 2010–2018) after propensity score matching.

	Prior MI			No Prior MI		
	Early	Late	*p* Value	Early	Late	*p* Value
	*n* = 2826	*n* = 2491		*n* = 6899	*n* = 4718	
Re-MI/angina (30 days)	131 (12.0)	102 (6.7)	<0.001	230 (9.0)	129 (4.2)	<0.001
MACE * (30 days)	593 (21.0)	261 (10.6)	<0.001	1119 (16.2)	413 (8.8)	<0.001
30-days mortality	182 (6.5)	110 (4.5)	0.003	362 (5.3)	155 (3.3)	<0.001
1-year mortality	409 (14.6)	250 (10.7)	<0.001	622 (9.1)	305 (6.8)	<0.001

*A composite of death, ACS, stroke, unstable angina, stent thrombosis, and urgent revascularization. Variables included in the model of propensity score (both the model for prior MI patients and for no prior MI patients): age, sex, dyslipidaemia, hypertension, diabetes mellitus, chronic kidney disease, history of heart failure, prior PVD, prior CABG, prior PCI, current smokers, family history of CAD. CABG—coronary artery bypass graft; CAD—coronary artery disease; PCI—percutaneous coronary; PVD—peripheral vascular disease; MACE—major adverse cardiac events; and MI—myocardial infarction.

**Table 5:** Clinical outcome of patients with and without prior MI admitted with STEMI vs. NSTE-ACS compared between time periods (early 2000–2008 vs. late 2010–2018).

	Prior MI						No Prior-MI					
	STEMI			NSTE-ACS			STEMI			NSTE-ACS		
	Early	Late	*p* Value	Early	Late	*p* Value	Early	Late	*p* Value	Early	Late	*p* Value
	*n* = 983	*n* = 711		*n* = 1838	*n* = 1780		*n* = 983	*n* = 711		*n* = 3153	*n* = 2468	
Re-MI/angina (30 days)	51 (15.8)	33 (7.7)	0.001	80 (10.4)	69 (6.3)	0.002	124 (9.7)	61 (4.1)	<0.001	106 (8.4)	68 (4.3)	<0.001
MACE *	239 (24.3)	91 (12.9)	<0.001	352 (19.2)	170 (9.7)	<0.001	646 (17.3)	210 (9.4)	<0.001	473 (15.0)	203 (8.3)	<0.001
(30 days)												
30-days mortality	96 (9.8)	47 (6.7)	0.032	85 (4.6)	63 (3.6)	0.155	256 (6.9)	96 (4.3)	<0.001	106 (3.4)	59 (2.4)	0.049
1-year mortality	160 (16.4)	88 (13.0)	0.068	246 (13.5)	162 (9.7)	0.001	373 (10.0)	157 (7.3)	0.001	249 (7.9)	148 (6.3)	0.023

* A composite of death, ACS, stroke, unstable angina, stent thrombosis, and urgent revascularization. MACE—major adverse cardiac events; MI—myocardial infarction; STEMI—ST elevation MI; and NSTE—ACS-non-ST elevation acute coronary syndrome.

## Data Availability

The data that support the findings of this study are available from the ACSIS registry organization, but restrictions apply to the availability of these data, which were used under license for the current study, and so are not publicly available. Data are, however, available from the authors upon reasonable request and with permission of the ACSIS registry organization.

## Supplementary Materials

The following are available online at https://www.mdpi.com/article/10.3390/jcm10235580/s1, Table S1: Baseline characteristics of patients with vs. without prior MI admitted with ACS (2000–2008), Table S2: Baseline characteristics, clinical presentation and management of patients with and without prior MI between time periods (early 2000–2008 vs. late 2010–2018) after propensity score matching, Table S3: prior MI vs No prior MI according to ESTEMI vs NSTE-ACS, Table S4: Baseline characteristics of patients with prior MI admitted with STEMI vs NSTE-ACS compared between time periods (early 2000–2008 vs late 2010–2018), Table S5: Clinical presentation and management of patients with prior MI admitted with STEMI vs NSTE-ACS comparing between time periods (early 2000–2008 vs late 2010–2018).
