# Supplementary material for: Temporal Trends and Outcome of Patients with Acute Coronary Syndrome and Prior Myocardial Infarction
Source: J Clin Med. 2021 Nov 27;10(23):5580. doi: 10.3390/jcm10235580 (PMC8658674; doi:10.3390/jcm10235580)
Supplement: Supplementary file 1 [file jcm-10-05580-s001.zip › Table S1.pdf]

Supplementary Materials:

Table S1: Baseline characteristics of patients with vs. without prior MI admitted with ACS (2000-2018)

	**No Prior MI (n=11617)**	**Prior MI (n=5317)**	**P value**
Age (years)	62.6 ±13.1	66.7±12.4	<0.001
Gender (male)	8757 (75.4)	4316 (81.2)	<0.001
Dyslipidemia	6735 (58.2)	4258 (80.1)	<0.001
Hypertension	6185 (53.4)	3877 (72.9)	<0.001
Current Smoker	4613 (39.7)	1701 (32.0)	<0.001
Diabetes mellitus	3641 (31.3)	2465 (46.4)	<0.001
Prior CABG	410 (3.5)	1251 (23.5)	<0.001
Prior PCI	1069 (9.2)	3709 (69.8)	<0.001
Chronic kidney disease	866 (7.5)	983 (18.5)	<0.001
PVD	661 (5.7)	744 (14.0)	<0.001
CVA/TIA	754 (6.5)	627 (11.9)	<0.001
**Baseline medications**			
Aspirin	3279 (32.7)	3753 (80.6)	<0.001
P_2Y_{12} inhibitors	375 (3.8)	985 (21.7)	<0.001
ACE-I/ARB	2278 (33.5)	2098 (65.0)	<0.001
Beta blockers	2399 (20.7)	2939 (55.3)	<0.001
Statins	3321 (28.6)	3289 (61.9)	<0.001
**Presentation and management**			
ST elevation at presentation	5993 (51.6)	1649 (31.9)	<0.001

Admission Killip III/IV	627 (5.5)	460 (8.9)	<0.001
**Revascularization therapy**			
Primary PCI	3808 (36.4)	949 (20.7)	<0.001
Any PCI	6269 (64.4)	2463 (53.9)	<0.001
CABG	520 (4.7)	200 (4.3)	0.217
Number of diseased vessels			
1	2868 (37.1)	742 (22.1)	<0.001
2	2427 (31.4)	1091 (32.4)	
3	1999 (25.9)	1424 (42.4)	
**In-hospital complications**			
Pulmonary edema (Killip-3)	636 (5.5)	474 (8.9)	<0.001
Cardiogenic shock (Killip-4)	371 (3.2)	203 (3.8)	0.039
Re-MI	132 (1.1)	91 (1.7)	0.003
Stent thrombosis (definite/probable/possible)	42 (0.6)	37 (1.0)	0.014
Free wall rupture	52 (0.4)	7 (0.1)	0.002
MR moderate – severe	202 (1.7)	123 (2.3)	0.013
Sustained VT (>125 bpm)	158 (1.4)	90 (1.7)	0.106
Primary VF	232 (2.0)	80 (1.5)	0.033
Acute renal failure	607 (5.2)	406 (7.7)	<0.001
**Treatment at discharge**			
Aspirin	10759 (94.7)	4845 (93.7)	0.010
P_2Y_{12} inh.	8289 (73.5)	3659 (71.5)	<0.001
Statins	9525 (84.4)	4459 (86.9)	0.001

ACE/ARB's	5926 (51.0)	2971 (55.9)	<0.001
Beta blockers	8755 (78.8)	4150 (82.3)	<0.001
Cardiac rehabilitation referral	3316 (52.2)	1357 (44.0)	<0.001
**Clinical outcome**			
Re- MI/angina (30 days)	359 (6.4)	233 (8.9)	<0.001
MACE (30 days)	1532 (13.2)	854 (16.2)	<0.001
30-days mortality	517 (4.5)	292 (5.6)	0.003
1-year mortality	927 (8.2)	659 (12.8)	<0.001
