# Supplementary material for: Temporal Trends and Outcome of Patients with Acute Coronary Syndrome and Prior Myocardial Infarction
Source: J Clin Med. 2021 Nov 27;10(23):5580. doi: 10.3390/jcm10235580 (PMC8658674; doi:10.3390/jcm10235580)
Supplement: Supplementary file 1 [file jcm-10-05580-s001.zip › Table S2.pdf]

Table S2: Baseline characteristics, clinical presentation and management of patients with and without prior MI between time periods (early 2000-2008 vs. late 2010-2018) after propensity score matching

	Prior MI			No prior MI		
	Early<br>n=1809	Late<br>n=1809	P value	Early<br>n=4490	Late<br>n=4490	P value
Age (years)	65.9 ±12.3	66.2±12.5	0.462	62.1±13.0	62.6±12.9	0.055
Sex (male)	1472 (81.4)	1460 (80.7)	0.641	3412 (76.0)	3387 (75.4)	0.555
Hypertension	1362 (75.3)	1364 (75.4)	0.969	2455 (54.7)	2509 (55.9)	0.261
Dyslipidemia	1516 (83.8)	1534 (84.8)	0.437	2827 (63.0)	2863 (63.8)	0.443
Diabetes mellitus	849 (46.9)	838 (46.3)	0.739	1410 (31.4)	1438 (32.0)	0.540
Family history of CAD	443 (24.5)	430 (23.8)	0.641	1239 (27.6)	1182 (26.3)	0.183
Active smoker	580 (32.1)	578 (32.0)	0.972	1846 (41.1)	1809 (40.3)	0.439
Chronic kidney disease	334 (18.5)	345 (19.1)	0.670	353 (7.9)	343 (7.6)	0.722
History of heart failure	357 (19.7)	335 (18.5)	0.375	133 (3.0)	128 (2.9)	0.802
Prior PVD	253 (14.0)	255 (14.1)	0.962	200 (4.5)	210 (4.7)	0.649
Prior PCI	1405 (77.7)	1391 (76.9)	0.606	361 (8.0)	376 (8.4)	0.590
Prior CABG	436 (24.1)	441 (24.4)	0.877	99 (2.2)	110 (2.4)	0.484
**Prior medications**						
Aspirin	1307 (85.3)	1372 (78.8)	<0.001	1217 (32.9)	1389 (32.9)	1.000
P_2Y_{12} inhibitors	301 (19.7)	460 (26.8)	<0.001	87 (2.3)	218 (5.3)	<0.001
ACE-I/ARB	376 (49.2)	752 (45.8)	0.136	417 (23.3)	973 (24.3)	0.398
Beta blockers	1003 (55.4)	1108 (61.2)	<0.001	918 (20.4)	985 (21.9)	0.088
Statins	1093 (60.4)	1311 (72.5)	<0.001	1164 (25.9)	1633 (36.4)	<0.001

**Revascularization therapy**						
Primary PCI	356 (19.7)	382 (21.1)	0.302	1578 (35.1)	1726 (38.4)	0.001
Any PCI	920 (50.9)	1121 (62.0)	<0.001	2650 (59.0)	3295 (73.4)	<0.001
CABG	87 (4.8)	47 (2.6)	0.001	259 (5.8)	152 (3.4)	<0.001
**Treatment at discharge**						
Aspirin	1656 (93.9)	1677 (94.5)	0.487	4116 (93.7)	4233 (96.2)	<0.001
P_2Y_{12} inhibitor	1066 (61.1)	1527 (86.2)	<0.001	2769 (63.5)	3896 (88.8)	<0.001
Statins	1479 (83.8)	1675 (95.6)	<0.001	3464 (79.1)	4149 (95.5)	<0.001
ACE/ARB's	703 (38.9)	1373 (75.9)	<0.001	1597 (35.6)	3323 (74.0)	<0.001
Beta blockers	1455 (82.6)	1436 (83.6)	0.453	3431 (78.3)	3355 (79.9)	0.079
Referral to cardiac rehabilitation	294 (35.0)	715 (47.9)	<0.001	738 (42.9)	2169 (58.7)	<0.001

ACE-I-angiotensin-converting enzyme inhibitor; ARB- angiotensin receptor blocker; CABG-coronary artery bypass graft; CAD- coronary artery disease; PCI- percutaneous coronary; PVD- peripheral vascular disease; MI-myocardial infarction.

Variables included in the model of propensity score (both the model for prior MI patients and for no prior MI patients): Age, Sex, Dyslipidemia , Hypertension , Diabetes mellitus, Chronic renal failure, History of heart failure, Prior PVD, Prior CABG, Prior PCI, current smokers, Family history of CAD.
