# Supplementary material for: Temporal Trends and Outcome of Patients with Acute Coronary Syndrome and Prior Myocardial Infarction
Source: J Clin Med. 2021 Nov 27;10(23):5580. doi: 10.3390/jcm10235580 (PMC8658674; doi:10.3390/jcm10235580)
Supplement: Supplementary file 1 [file jcm-10-05580-s001.zip › Table S3.pdf]

Table S3: prior MI vs No prior MI according to STEMI vs NSTE-ACS

	Prior MI			No prior MI		
	**NSTE-ACS n=3618**	**STEMI n=1694**	**P value**	**NSTE-ACS n=5621**	**STEMI n=5993**	**P value**
Age (years)	67.6 ±12.2	64.7±12.5	<0.001	64.1 ±12.8	61.3± 13.2	<0.001
Gender (male)	2909 (80.4)	1403 (82.8)	0.039	4071 (72.4)	4684 (78.2)	<0.001
Dyslipidemia	2997 (82.8)	1259 (74.3)	<0.001	3635 (64.7)	3101 (51.7)	<0.001
Hypertension	2780 (76.8)	1095 (64.6)	<0.001	3439 (61.3)	2744 (45.9)	<0.001
Active Smoker	1000 (27.6)	700 (41.3)	<0.001	1838 (32.9)	2773 (46.4)	<0.001
Diabetes mellitus	1761 (48.7)	701 (41.4)	<0.001	2006 (35.7)	1633 (27.2)	<0.001
Prior CABG	1033 (28.6)	218 (12.9)	<0.001	331 (5.9)	79 (1.3)	<0.001
Prior PCI	2554 (70.6)	1155 (68.2)	0.076	792 (14.1)	276 (4.6)	<0.001
Chronic kidney disease	776 (21.4)	206 (12.2)	<0.001	554 (9.9)	311 (5.2)	<0.001
PVD	533 (14.7)	209 (12.3)	0.021	405 (7.2)	255 (4.3)	<0.001
Stroke/TIA	437 (12.1)	189 (11.2)	0.361	401 (7.1)	351 (5.9)	0.006
History of heart failure	787 (21.9)	233 (13.8)	<0.001	265 (4.7)	82 (1.4)	<0.001
Aspirin	2657 (82.6)	1093 (76.2)	<0.001	2115 (42.6)	1163 (22.9)	<0.001
P_2Y_{12} inhibitors	785 (25.0)	200 (14.3)	<0.001	288 (5.8)	87 (1.7)	<0.001
ACE-I/ ARB	1553 (69.4)	545 (54.9)	0.002	1523 (41.7)	855 (25.3)	<0.001
Beta blockers	2178 (69.2)	760 (55.0)	<0.001	1599 (33.1)	800 (16.1)	<0.001
Statins	2408 (77.1)	880 (63.8)	<0.001	2096 (44.8)	1224 (25.7)	<0.001

**Revascularization therapy**						
Primary PCI	32 (1.0)	916 (63.2)	<0.001	112 (2.0)	4119 (68.7)	<0.001
Any PCI	1700 (47.0)	1163 (68.7)	<0.001	3018 (53.7)	4458 (74.4)	<0.001
CABG	156 (4.3)	71 (4.2)	0.910	363 (8.2)	185 (4.0)	<0.001
**In-hospital complications**						
Pulmonary edema (Killip-3)	325 (9.0)	145 (8.6)	0.656	300 (5.3)	336 (5.6)	0.545
Cardiogenic shock (Killip-4)	82 (2.3)	121 (7.2)	<0.001	80 (1.4)	291 (4.9)	<0.001
Re-MI	52 (1.4)	39 (2.3)	0.032	51 (0.9)	81 (1.4)	0.029
Stent thrombosis	12 (0.5)	25 (2.4)	<0.001	5 (0.1)	37 (1.0)	<0.001
Free wall rupture	1 (0.0)	6 (0.4)	0.008	3 (0.1)	49 (0.8)	<0.001
MR moderate – severe	81 (2.2)	41 (2.4)	0.758	101 (1.8)	101 (1.7)	0.697
Sustained VT (>125 bpm)	50 (1.4)	40 (2.4)	0.014	40 (0.7)	118 (2.0)	<0.00
Primary VF	21 (0.6)	59 (3.5)	<0.001	21 (0.4)	211 (3.5)	<0.001
Acute renal failure	281 (7.8)	125 (7.4)	0.650	275 (4.9)	332 (5.6)	0.127
**Treatment at discharge**						
Aspirin	3313 (93.7)	1528 (93.7)	0.996	5211 (94.2)	5545 (95.2)	0.022
P_2Y_{12} inh.	2486 (71.0)	1172 (72.7)	0.230	3821 (69.7)	4466 (77.0)	<0.001
Statins	3088 (87.9)	1370 (84.8)	0.003	4692 (85.6)	4827 (83.3)	0.001
ACE/ARB's	2010 (55.6)	958 (56.6)	0.514	2745 (48.8)	3181 (53.1)	<0.001
Beta blockers	2860 (82.9)	1287 (80.9)	0.091	4156 (77.2)	4597 (80.2)	<0.001
Referral to cardiac rehabilitation	877 (39.8)	480 (54.5)	<0.001	1492 (46.0)	1824 (58.8)	<0.001
**Clinical outcome**						

Re- MI/angina (30 days)	149 (8.0)	84 (11.2)	0.012	174 (6.1)	185 (6.7)	0.365
MACE* (30 days)	522 (14.6)	330 (19.5)	<0.001	676 (12.1)	856 (14.3)	<0.001
30-days mortality	148 (4.2)	143 (8.5)	<0.001	165 (3.0)	352 (5.9)	<0.001
1-year mortality	408 (11.7)	248 (15.0)	0.001	397 (7.2)	530 (9.0)	<0.001

*a composite of death, ACS, stroke, unstable angina, stent thrombosis, and urgent revascularization

ACE-I-angiotensin-converting enzyme inhibitor; ARB- angiotensin receptor blocker; CABG-coronary artery bypass graft; PCI- percutaneous coronary; PVD- peripheral vascular disease; TIA- transient ischemic attack; MACE- major adverse cardiac events; MI- myocardial infarction; MR- mitral regurgitation; VF- ventricular fibrillation; VT-ventricular tachycardia
