# Supplementary material for: Temporal Trends and Outcome of Patients with Acute Coronary Syndrome and Prior Myocardial Infarction
Source: J Clin Med. 2021 Nov 27;10(23):5580. doi: 10.3390/jcm10235580 (PMC8658674; doi:10.3390/jcm10235580)
Supplement: Supplementary file 1 [file jcm-10-05580-s001.zip › Table S4.pdf]

Table S4: Baseline characteristics of patients with prior MI admitted with STEMI vs NSTE-ACS compared between time periods (early 2000-2008 vs late 2010-2018)

	STEMI			NSTE-ACS		
	**Early n=983**	**Late n=711**	**P value**	**Early n=1838**	**Late n=1780**	**P value**
Age (years)	64.7 ±12.8	64.7 ±12.1	0.963	67.6 ±12.4	67.5 ±11.9	0.771
Gender (male)	790 (80.4)	613 (86.2)	0.002	1450 (78.9)	1459 (82.0)	0.022
Dyslipidemia	648 (65.9)	611 (85.9)	<0.001	1400 (76.2)	1597 (89.7)	<0.001
Hypertension	563 (57.3)	532 (74.8)	<0.001	1287 (70.0)	1493 (83.9)	<0.001
Active Smoker	386 (39.3)	314 (44.2)	0.049	418 (22.7)	582 (32.7)	<0.001
Diabetes mellitus	384 (39.1)	317 (44.6)	0.026	799 (43.5)	962 (54.0)	<0.001
Prior CABG	127 (12.9)	91 (12.8)	1.000	570 (31.0)	463 (26.0)	0.001
Prior PCI	541 (55.0)	614 (86.4)	<0.001	1096 (59.6)	1458 (81.9)	<0.001
Chronic kidney disease	113 (11.5)	93 (13.1)	0.363	405 (22.0)	371 (20.8)	0.405
PVD	129 (13.1)	80 (11.3)	0.280	301 (16.4)	232 (13.0)	0.005
Stroke/TIA	110 (11.3)	79 (11.1)	0.985	401 (7.1)	351 (5.9)	0.006
History of heart failure	138 (14.0)	95 (13.4)	0.743	209 (11.5)	228 (12.8)	0.243
**Baseline medications**						
Aspirin	581 (77.2)	512 (75.1)	0.388	1296 (84.5)	1361 (80.8)	0.007
P_2Y_{12} inhibitors	78 (10.4)	122 (18.9)	<0.001	278 (18.2)	507 (31.4)	<0.001
ACE-I/ARB	184 (48.9)	361 (58.7)	<0.001	446 (61.5)	1107 (73.4)	<0.001
Beta blockers	384 (51.1)	376 (59.6)	0.002	1028 (67.4)	1150 (70.9)	0.037
Statins	419 (55.8)	461 (73.4)	<0.001	1047 (68.7)	1361 (85.2)	<0.001

* Values are presented as number (%) or mean ± standard deviation.

ACE-I-angiotensin-converting enzyme inhibitor; ARB- angiotensin receptor blocker; CABG-coronary artery bypass graft; NSTE-ACS- non-ST elevation acute coronary syndrome; PCI-percutaneous coronary; PVD- peripheral vascular disease; STEMI-ST elevation MI; TIA-transient ischemic attack
