# Supplementary material for: Temporal Trends and Outcome of Patients with Acute Coronary Syndrome and Prior Myocardial Infarction
Source: J Clin Med. 2021 Nov 27;10(23):5580. doi: 10.3390/jcm10235580 (PMC8658674; doi:10.3390/jcm10235580)
Supplement: Supplementary file 1 [file jcm-10-05580-s001.zip › Table S5.pdf]

Table S5: Clinical presentation and management of patients with prior MI admitted with STEMI vs NSTE-ACS comparing between time periods (early 2000-2008 vs late 2010-2018)

	STEMI			NSTE-ACS		
	**Early**<br>**n=983**	**Late**<br>**n=711**	**P value**	**Early**<br>**n=1838**	**Late**<br>**n=1780**	**P value**
**Revascularization therapy**						
Primary PCI	534 (54.3)	535 (75.2)	<0.001	28 (1.5)	4 (0.2)	<0.001
Any PCI	577 (58.7)	586 (82.4)	<0.001	748 (40.7)	952 (53.5)	<0.001
CABG	62 (6.4)	9 (1.3)	0.334	94 (5.1)	62 (3.5)	0.018
**In-hospital complications**						
Pulmonary edema (Killip-3)	106 (10.8)	39 (5.5)	<0.001	214 (11.7)	111 (6.2)	<0.001
Cardiogenic shock (Killip-4)	82 (8.4)	39 (5.5)	0.029	54 (3.0)	28 (1.6)	0.008
Re-MI	31 (3.2)	8 (1.1)	0.009	35 (1.9)	17 (1.0)	0.022
Stent thrombosis	14 (4.0)	11 (1.5)	0.023	5 (0.6)	7 (0.4)	0.646
Free wall rupture	5 (0.5)	1 (0.1)	0.396	1 (0.1)	0 (0.0)	1.000
MR moderate – severe	33 (3.4)	8 (1.1)	0.005	50 (2.7)	31 (1.7)	0.058
Sustained VT (>125 bpm)	31 (3.2)	9 (1.3)	0.018	27 (1.5)	23 (1.3)	0.740
Primary VF	41 (4.2)	18 (2.5)	0.090	14 (0.8)	7 (0.4)	0.211
Acute renal failure	75 (7.7)	50 (7.1)	0.712	176 (9.7)	105 (5.9)	<0.001
**Treatment at discharge**						
Aspirin	871 (92.3)	657 (95.8)	0.005	1665 (92.4)	1648 (95.0)	0.002
P_2Y_{12} inhibition	549 (59.2)	623 (90.9)	<0.001	976 (55.1)	1510 (87.4)	<0.001
Statins	724 (77.2)	646 (95.3)	<0.001	1419 (79.3)	1669 (96.8)	<0.001
ACE/ARB's	408 (41.5)	550 (77.4)	<0.001	666 (36.2)	1344 (75.5)	<0.001

Beta blockers	735 (78.4)	552 (84.7)	0.002	1445 (80.6)	1415 (85.4)	<0.001
Referral to cardiac rehabilitation	136 (44.4)	344 (59.8)	<0.001	214 (28.6)	663 (45.5)	<0.001

ACE-I-angiotensin-converting enzyme inhibitor; ARB- angiotensin receptor blocker; CABG-coronary artery bypass graft; NSTE-ACS- non-ST elevation acute coronary syndrome; PCI-percutaneous coronary; PVD- peripheral vascular disease; TIA- transient Ischemic attack; STEMI-ST elevation MI; MACE- major adverse cardiac events; MI- myocardial infarction; MR- mitral regurgitation; VF- ventricular fibrillation; VT-ventricular tachycardia
